# Predictive value of procalcitonin decrease in patients with severe sepsis: a prospective observational study

**DOI:** 10.1186/cc9327

**Published:** 2010-11-15

**Authors:** Sari Karlsson, Milja Heikkinen, Ville Pettilä, Seija Alila, Sari Väisänen, Kari Pulkki, Elina Kolho, Esko Ruokonen

**Affiliations:** 1 Department of Intensive Care Medicine, Tampere University Hospital, Teiskontie 35, 33521 Tampere, Finland; 2 Department of Clinical Chemistry, University of Eastern Finland and Eastern Finland Laboratory Centre, Puijonlaaksontie 2, 70211 Kuopio, Finland; 3 Division of Anaesthesia and Intensive Care Medicine, Department of Surgery, Helsinki University Hospital, Haartmaninkatu 4, 00029 HUS, Helsinki, Finland; 4 Department of Anaesthesia and Intensive Care Medicine, Kymenlaakso Central Hospital, Kotkantie 41, 48210 Kotka, Finland; 5 Division of Infectious Diseases, Department of Medicine, Helsinki University Hospital, Haartmaninkatu 4, 00029 HUS, Helsinki, Finland; 6 Department of Intensive Care Medicine, Kuopio University Hospital, Puijonlaaksontie 2, 70211 Kuopio, Finland

## Abstract

**Introduction:**

This prospective study investigated the predictive value of procalcitonin (PCT) for survival in 242 adult patients with severe sepsis and septic shock treated in intensive care.

**Methods:**

PCT was analyzed from blood samples of all patients at baseline, and 155 patients 72 hours later.

**Results:**

The median PCT serum concentration on day 0 was 5.0 ng/ml (interquartile range (IQR) 1.0 and 20.1 ng/ml) and 1.3 ng/ml (IQR 0.5 and 5.8 ng/ml) 72 hours later. Hospital mortality was 25.6% (62/242). Median PCT concentrations in patients with community-acquired infections were higher than with nosocomial infections (P = 0.001). Blood cultures were positive in 28.5% of patients (*n *= 69), and severe sepsis with positive blood cultures was associated with higher PCT levels than with negative cultures (P = < 0.001). Patients with septic shock had higher PCT concentrations than patients without (P = 0.02). PCT concentrations did not differ between hospital survivors and nonsurvivors (P = 0.64 and P = 0.99, respectively), but mortality was lower in patients whose PCT concentration decreased > 50% (by 72 hours) compared to those with a < 50% decrease (12.2% vs. 29.8%, P = 0.007).

**Conclusions:**

PCT concentrations were higher in more severe forms of severe sepsis, but a substantial concentration decrease was more important for survival than absolute values.

## Introduction

Because promptly administered antimicrobial and early goal-directed treatment has been shown to improve outcome in patients with severe sepsis [1,2], early recognition of infection as a cause of critical illness is of major importance. Various biomarkers, such as C-reactive protein (CRP), interleukin-6 (IL-6), and triggering receptor expressed on myeloid cells-1 (TREM-1), have been studied as a means of detecting infection as a cause of systemic inflammation response syndrome, but none has been shown to be used reliably to diagnose sepsis [3]. In addition, CRP and other biomarkers have not been shown to detect patients with a high risk of poor outcome [4].

Procalcitonin (PCT) is a 116-amino acid prohormone of calcitonin [5] that is found in the bloodstream without changes in the total amount of calcitonin [6]. The production of PCT is stimulated by inflammatory cytokines, such as tumor necrosis factor-alpha and IL-6 [7]. PCT concentrations increase after bacterial infection but also in noninfectious conditions with systemic inflammation, such as multiple trauma, cardiogenic shock, induction of hypothermia after cardiac arrest, and drug sensitivity reactions [8-11]. PCT concentrations are also elevated after major surgery [12]. However, bacterial infections increase the expression of the PCT-producing *CALC-1 *gene in multiple extrathyroid tissues throughout the body [13].

Patients without infection and inflammation usually have low serum PCT concentrations (< 0.05 ng/mL). In patients with severe sepsis or septic shock, PCT concentrations may increase significantly (up to 1,000 ng/mL) [5]. The cutoff value for sepsis has been set at 0.44 to 1.0 ng/mL in different studies [14,15]. PCT concentrations have been used to differentiate noninfected patients from infected patients in prospective clinical studies, and higher mortality has been associated with patients who have increasing or persistently high PCT concentrations [16]. Recent studies concerning PCT have focused on patients with suspected or verified bacterial infections, and the duration of antibiotic treatment was guided by decreasing PCT concentrations [17-19]. Reduced antibiotic administration without increased adverse outcomes has been shown in patients with lower respiratory tract infections (LRTIs) [18], medical intensive care unit (ICU) patients [19], and patients with severe sepsis and septic shock [20].

Meta-analyses of PCT have produced conflicting results. One study concluded that PCT measurement cannot differentiate sepsis reliably from other causes of systemic inflammatory response syndrome and should not be used widely in a critical care setting [21]. In contrast, another study regarded PCT as superior to CRP measurement and concluded that PCT should be used to diagnose sepsis in ICUs [22]. Differences in the case mix may contribute to the varying results in critical care settings: on admission to the hospital or ICU, patients are at different phases in the course of their sepsis; preceding antibiotic treatment may be absent, ineffective [23], or delayed [1]; and in postoperative patients, the type of surgery may influence PCT concentrations [24].

In the present study, we measured PCT concentrations twice in adult ICU patients with clinically diagnosed severe sepsis in the first 3 days after diagnosis. We evaluated PCT concentrations and the type of organ dysfunction, the type of infection (blood culture-positive, community-acquired, or nosocomial), and the predictive value for outcome of the first PCT concentration and the decrease in PCT after treatment in this large population of patients with severe sepsis.

## Materials and methods

### Patient selection

This study was part of the Finnsepsis study, a prospective observational cohort study of incidence and outcome of severe sepsis in Finland [25]. All adult consecutive ICU admission episodes (4,500) in 24 ICUs were screened for severe sepsis in a 4-month period (from 1 November 2004 to 28 February 2005). Patients were eligible if they fulfilled the American College of Chest Physicians/Society of Critical Care Medicine (ACCP/SCCM) criteria for severe sepsis or septic shock [26]. Study entry (day 0) was the time when these criteria were first met. Consent from the ethics committee was granted from each hospital. All patients or their next of kin gave written consent for the study. APACHE II (Acute Physiology and Chronic Health Evaluation II) score and SAPS II (Simplified Acute Physiology Score II) [27,28], organ dysfunction evaluated with SOFA (Sequential Organ Failure Assessment) score, maximum SOFA scores [29,30], and ICU and hospital mortalities were recorded. Septic shock was defined as cardiovascular SOFA score 4, and acute kidney injury was defined as renal SOFA score 3 or 4. Severe sepsis was defined as community-acquired if the infection was present or suspected at hospital admission or less than 48 hours thereafter and was defined as nosocomial if the infection was diagnosed at least 48 hours after hospital admission. Blood CRP concentrations were analyzed as daily routine samples in each participating hospital. Blood cultures were drawn when clinically indicated and were analyzed locally.

### Blood samples

Arterial blood samples for PCT analyses were drawn after informed consent within 24 hours of study entry (day 0) and 72 hours thereafter. The reason for exclusion was failure to obtain consent. Blood for serum samples was collected, and the samples were prepared within 60 minutes of sampling. The samples were stored at -80°C for later analysis. Serum PCT levels were measured with the Cobas 6000 analyzer (Hitachi High-Technologies Corporation, Tokyo, Japan). Analyzer reagents (Elecsys B·R·A·H·M·S PCT assay) were developed in collaboration with B·R·A·H·M·S Aktiengesellschaft (Hennigsdorf, Germany) and Roche Diagnostics (Mannheim, Germany). The functional assay sensitivity (that is, the lowest concentration that can be quantified with a between-run imprecision of 20%) met the Roche Diagnostics specification of 0.06 ng/mL. The respective within- and between-day coefficients of variation for PCT analyses were 1.4% and 3.0% for 0.46 ng/mL PCT and 1.1% and 2.6% for 9.4 ng/mL PCT.

### Statistical analyses

Data are presented as median and interquartile range (IQR) (25th to 75th percentiles), absolute value and percentage, or mean ± standard deviation. The nonparametric data between survivors and nonsurvivors were compared with the Mann-Whitney *U *test, and categorical variables were compared with the chi-square test. PCT kinetics are expressed as delta PCT (ΔPCT) concentrations. ΔPCT was calculated as the difference between concentrations on day 0 and 72 hours (day 0 to 72 hours). ΔPCT was positive with decreasing concentrations and negative with increasing concentrations. The level of change between the two samples (for example, greater than 50%) was calculated as a proportion of ΔPCT/PCT on day 0. The sensitivity, specificity, and positive likelihood ratio for different PCT cutoff levels were calculated. To determine the prognostic accuracy of PCT and CRP on both time points, receiver operating characteristic (ROC) curves were constructed and the areas under the curve (AUCs) were calculated with 95% confidence intervals (CIs). A *P *value of less than 0.05 was considered to be statistically significant in all tests. The analyses were performed using SPSS 17.0 software (SPSS Inc., Chicago, IL, USA).

## Results

Informed consent and blood samples for the PCT analyses were obtained from 242 out of 470 patients (51.2%) of the Finnsepsis study population. Two hundred forty-two samples were obtained at baseline (day 0); of these, 155 samples were available 72 hours later. Fourteen patients died and 13 were discharged from the ICU before the second sample was obtained. Owing to logistical reasons, an additional 59 samples were not available.

**Figure 1:**
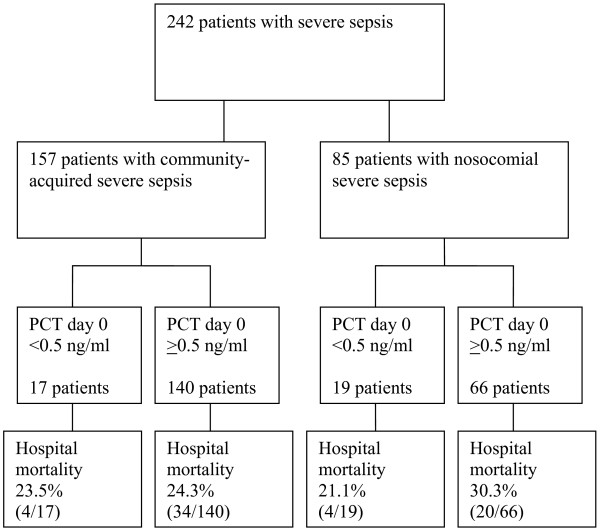
**Flowchart of the study**. PCT, procalcitonin.

The flowchart of the study is presented in Figure 1. The patients were divided by the type of infection and the cutoff concentration for PCT to detect unlikely sepsis (< 0.5 ng/mL) in semiquantitative PCT measurements (PCT-Q test) [31]. Age, gender, APACHE II score, SAPS II, maximum SOFA score, ICU mortalities, and hospital mortalities did not differ from the Finnsepsis patients who did not have PCT analyses (*P *= 0.75, 0.63, 0.58, 0.35, 0.22, 024, and 0.18, respectively). The infection and mortality data of patients with community-acquired or nosocomial severe sepsis are presented in Table 1. Mortality in patients with positive blood cultures did not differ from patients with blood culture-negative infections (26.1% and 25.4%, respectively; *P *= 0.92). Hospital mortality of patients with severe septic shock (cardiovascular SOFA score 4) was higher than that of patients with less severe or absent cardiovascular failure (31.6% versus 22.4%, *P *= 0.015).

**Table 1:** Patient data for all study patients and different types of infections

	All patients	Community-acquired	Nosocomial	*P *value
Number of patients	242	157/242 (64.9%)	85/242 (35.1%)	< 0.001
Age in years (SD)	59.8 (15.4)	58.2 (15.6)	62.7 (14.7)	0.03
Males (percentage)	165 (68.2%)	109 (69.4%)	56 (65.9%)	0.57
APACHE II score (SD)	24.0 (9.0)	23.9 (8.8)	24.1 (9.5)	0.93
SAPS II (SD)	43.8 (16.8)	42.6 (16.0)	46.1 (17.9)	0.22
SOFA on day 1^a ^(SD)	8.4 (3.6)	8.5 (3.6)	8.2 (3.5)	0.74
SOFAmax^b ^(SD)	10.9 (4.3)	11.0 (4.4)	10.7 (4.1)	0.68
Postoperative (percentage)	63 (26.0%)	31 (19.7%)	32 (37.6%)	< 0.01
Chronic renal failure	4 (1.7%)	1 (0.6%)	3 (3.5%)	0.16
Chronic lung disease	25 (10.3%)	17 (10.8%)	8 (9.4%)	0.84
Chronic hepatic disease	13 (5.4%)	6 (3.8%)	7 (8.2%)	0.22
Immunosuppression	30 (12.4%)	20 (12.7%)	10 (11.7%)	0.80
ICU mortality	33/242 (13.6%)	20/157 (12.7%)	13/85 (15.3%)	0.58
Hospital mortality	62/242 (25.6%)	38/157 (24.2%)	24/85 (28.2%)	0.49
				
Source of infection				
Pulmonary	101 (41.7%)	69 (43.9%)	32 (37.6%)	0.34
Intra-abdominal	77 (31.9%)	42 (26.8%)	35 (41.2%)	0.02
Skin or soft tissue	24 (9.9%)	17 (10.8%)	7 (8.2%)	0.52
Urinary tract	11 (4.5%)	8 (5.1%)	3 (3.5%)	0.58
Other	33 (13.6%)	24 (15.3%)	9 (10.6%)	0.31
				
Blood cultures				
Blood cultures taken	160/242 (66.1%)	110/157 (70.1%)	49/85 (57.6%)	
Positive blood cultures	69/160 (43.1%)	56/110 (50.9%)	13/49 (26.5%)	
				
Microbes in positive blood cultures				
*Streptococcus pneumoniae*	13	13	0	
*Staphylococcus aureus*	11	10	1	
*Streptococcus *species	9	9	0	
Other Gram-positive	4	4	0	
*Escherichia coli*	14	11	3	
Other Gram-negative	13	8	5	
Yeasts	4	1	3	
Mycobacterium	1	0	1	
				
Ongoing antibiotic treatment before day 0	98/242 (40.5%)	38 (24.2%)	60 (70.6%)	< 0.001

*P *values refer to patients with community-acquired or nosocomial infections. ^a^Sequential Organ Failure Assessment score on the day after study entry. ^b^Maximum Sequential Organ Failure Assessment score. APACHE II, Acute Physiology and Chronic Health Evaluation II; ICU, intensive care unit; SAPS II, Simplified Acute Physiology Score II; SD, standard deviation.

### Procalcitonin concentrations

The median PCT concentrations in patients with severe sepsis are presented in Table 2. On day 0, the range varied from 0.02 to 261.9 ng/mL, and after 72 hours, the range varied from 0.03 to 439 ng/mL. PCT concentrations did not differ between hospital survivors and nonsurvivors at either time point (*P *= 0.64 and *P *= 0.99 for day 0 and 72 hours, respectively). The ROC curves for day-0 and 72-hour PCT concentrations and mortality showed AUCs of 0.42 (95% CI 0.31 to 0.54, *P *= 0.19) and 0.50 (95% CI 0.38 to 0.62, *P *= 0.99), respectively. High PCT concentrations (PCT > 10 ng/mL) on day 0 or 72 hours did not predict mortality; AUCs were 0.58 (CI 0.43 to 0.73, *P *= 0.25) and 0.36 (CI 0.09 to 0.62, *P *= 0.33), respectively.

**Table 2:** Procalcitonin concentrations in different patient groups

	Procalcitonin, ng/mL	
	Day 0	72 hours
All patients	5.0 (1.0-20.1)	1.3 (0.5-5.8)
Septic shock (SOFA 4)^a^	6.5 (1.6-29.0)	2.3 (0.7-7.4)
Without septic shock (SOFA 0-3)^a^	3.2 (0.9-14.7)	1.1 (0.3-4.4)
Severe acute kidney injury (SOFA 3-4)^b^	9.4 (2.4-38.2)	4.9 (0.9-9.5)
Without severe acute kidney injury (SOFA 0-2)^b^	4.3 (0.9-16.4)	1.2 (0.3-4.9)
Blood culture-positive infection^c^	15.6 (4.3-43.6)	5.2 (1.7-8.7)
Blood culture-negative infection^c^	2.9 (0.8-12.5)	1.0 (0.3-4.3)
Community-acquired infection^d^	6.6 (1.4-33.2)	2.4 (0.7-6.5)
Nosocomial infection^d^	2.9 (0.8-10.6)	0.9 (0.2-2.8)

The data are presented as median (interquartile range). *P *values refer to differences between patient groups (for example, those with and those without septic shock). ^a^*P *= 0.020 on day 0 and *P *= 0.031 at 72 hours; ^b^*P *= 0.027 on day 0 and *P *= 0.02 at 72 hours; ^c^*P *< 0.001 on day 0 and *P *< 0.001 at 72 hours; ^d^*P *= 0.001 on day 0 and *P *= 0.003 at 72 hours. SOFA, Sequential Organ Failure Assessment.

### Procalcitonin and type of infection

**Figure 2:**
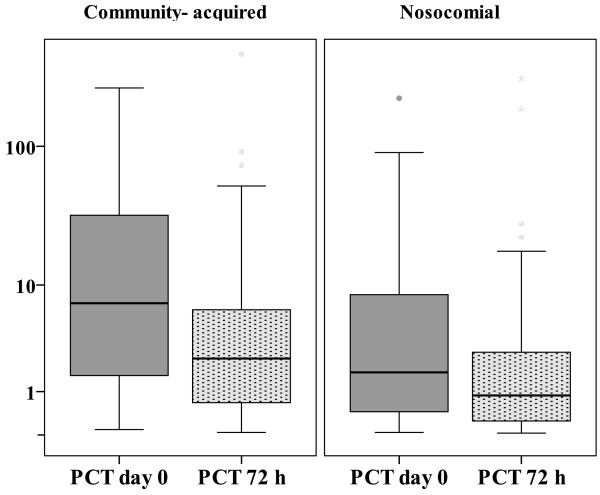
**Procalcitonin (PCT) concentrations in patients with community-acquired or nosocomial infections**. *P *= 0.001 on day 0 and *P *= 0.003 at 72 hours between the patient groups. PCT concentrations are shown in logarithmic scale and are presented in nanograms per milliliter.

**Figure 3:**
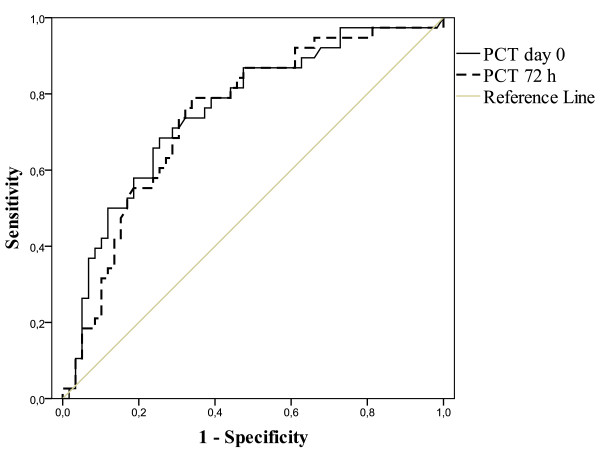
**Receiver operating characteristic curve for procalcitonin (PCT) concentration and positive blood culture**. Areas under the curve are 0.76 (95% confidence interval [CI] 0.66 to 0.86, *P *< 0.001) for PCT on day 0 and 0.74 (95% CI 0.64 to 0.84, *P *< 0.001) for PCT at 72 hours.

The median PCT concentrations on day 0 and after 72 hours in patients with community-acquired infections were higher than in patients with nosocomial infections (*P *= 0.001 and *P *= 0.003, respectively) (Figure 2). Blood cultures were drawn from 160 out of 242 patients (66%) and were positive in 69 out of 242 (28.5%). PCT concentrations in relation to blood cultures and community-acquired or nosocomial infections are presented in Table 2. PCT concentrations were higher in patients with positive blood cultures at both time points (*P *< 0.001 and *P *< 0.001, respectively). The ROC curves for day-0 and 72-hour PCT concentrations predicted blood culture-positive infections, with AUCs of 0.76 (95% CI 0.66 to 0.86, *P *< 0.001) and 0.74 (95% CI 0.64 to 0.84, *P *< 0.001) (Figure 3). The cutoff PCT concentration for blood culture-positive infection with 90% sensitivity (95% CI 83% to 97%) was 1.2 ng/mL. The positive likelihood ratio was 1.4 (95% CI 1.2 to 1.6). The cutoff PCT concentration of 10 ng/mL had 62% (95% CI 51% to 74%) sensitivity and 73% (95% CI 63% to 82%) specificity with a positive likelihood ratio of 2.3 (95% CI 1.5 to 3.3) for positive blood culture. PCT of greater than 20 ng/mL had 85% specificity (95% CI 77% to 92%), and the positive likelihood ratio was 3 (95% CI 1.7 to 5.2).

Thirty-six patients with clinically diagnosed severe sepsis and low PCT concentrations ('sepsis unlikely') had median PCT concentrations of 0.17 ng/mL (IQR 0.93 and 0.27 ng/mL) on day 0 and 0.13 ng/mL (IQR 0.08 and 0.22 ng/mL). Only one patient had a strongly increasing PCT of 17.88 ng/mL after 72 hours. The patient had an intra-abdominal infection. Nosocomial infection was found in 53% (19/36) of these patients, and the sources of infection were the lungs in 44% (16/36) and intra-abdominal in 31% (11/36). One patient had a blood culture-positive infection, and 14 other patients had significant microbial growths.

### Procalcitonin and organ dysfunction

Patients with septic shock or acute kidney injury also had significantly higher PCT concentrations on day 0 compared with patients with milder or absent organ dysfunction (*P *= 0.020 and *P = *0.027, respectively) (Table 2). When patients with two available PCT samples (*n *= 155) were divided into two groups according to decreasing PCT (*n *= 130) or increasing PCT (*n *= 25), no significant differences were found in organ dysfunction (*P *= 0.58).

### Changes in procalcitonin concentrations

We analyzed the difference in PCT concentrations on day 0 and 72 hours (ΔPCT) for the 155 patients with two blood samples available. The PCT concentration decreased in 130 patients and increased in the remaining 25 patients, but the change in PCT concentration was not associated with mortality (*P *= 0.25). Of the patients with decreasing PCT concentrations, 66% (86/130) had community-acquired infections and 34% (44/130) had nosocomial infections (*P *= 0.014).

**Figure 4:**
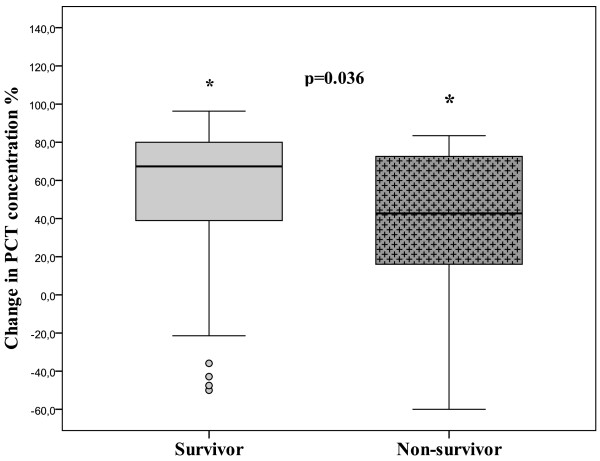
**Change in procalcitonin (PCT) concentration (ΔPCT/PCT on day 0) in hospital survivors and nonsurvivors**. Asterisks refer to difference in PCT change. Positive change is defined as decreasing concentrations.

When the decreases in PCT concentrations were divided into arbitrary classes from greater than 50% to greater than 90%, a substantial decrease in PCT concentration of greater than 50% between the first and second time points had an effect on hospital survival (Figure 4). The hospital mortality in patients with a greater than 50% decrease in PCT was 12.2% (12/98) compared with 29.8% (17/57) in patients with a less than 50% decrease (*P *= 0.007). Community-acquired infections (69.8%, 67/96) were associated with a greater than 50% decrease more often than nosocomial infections were (52.5%, 31/59; *P *= 0.031). In patients with community-acquired severe sepsis, a greater than 50% decrease was associated with better outcome (62.5% survivors) compared with patients with less than 50% decrease (19.8% survivors, *P *= 0.05). However, this association was not present for patients with nosocomial severe sepsis (*P *= 0.40). In all patients with available ΔPCT (*n *= 155), a greater than 50% PCT decrease showed a poor AUC of 0.52 (95% CI 0.36 to 0.68). The PCT decrease of greater than 50% was not independently associated with in-hospital mortality (*P *= 0.47, odds ratio 0.99, 95% CI 0.96 to 1.02) either.

### C-reactive protein measurements

The median CRP concentrations of this study population were 197 mg/L (104 and 294 mg/L) on day 0 and 149 mg/L (76 and 201 mg/L) after 72 hours. Patients with positive blood cultures had higher day-0 CRP concentrations compared with patients with negative cultures (244 mg/L [131 to 325 mg/mL] and 187 mg/L [89 to 273 mg/L], respectively; *P *= 0.016). For patients with decreasing or increasing PCT concentrations, the CRP levels did not differ significantly on day 0 or after 72 hours (*P *= 0.138 and *P *= 0.552, respectively). CRP concentrations were not associated with the severity of cardiovascular dysfunction (*P *= 0.35 and *P *= 0.11 for day 0 and 72 hours, respectively). The ROC curves for day-0 and 72-hour CRP concentrations and mortality showed inadequate AUCs of 0.52 (95% CI 0.46 to 0.58) and 0.59 (95% CI 0.53 to 0.65), respectively (*P *= 0.99).

## Discussion

PCT concentrations varied largely among individual ICU patients with clinically diagnosed severe sepsis. The predictive value of the individual PCT samples for mortality was poor, but a prompt 50% decrease in PCT indicating resolving infection was associated with a favorable outcome. Patients with community-acquired infections had higher PCT concentrations compared with patients with nosocomial infections. PCT concentrations were not superior to CRP concentrations for predicting mortality or severity of illness in our study.

The high values (up to 439 ng/mL) of the PCT concentrations in this study are in accordance with those in other studies [6,15]. The method used in this study was able to detect low PCT concentrations (sensitivity of 0.06 ng/mL) more sensitively than the older LUMItest assay (B·R·A·H·M·S), which has a detection limit of 0.3 to 0.5 ng/mL [32] and was used in many previous studies [15,16]. The cutoff limit for PCT is often set at approximately 1 ng/mL in studies detecting sepsis from other causes of systemic inflammatory response [15,16,33,34]. The median PCT concentrations in our patients were 5.0 ng/mL on the day that severe sepsis was diagnosed and 6.5 ng/mL in patients with septic shock. These concentrations are concordant with other studies in patients with diagnosed severe sepsis [20,35]. In our study, as many as 22.7% of patients (55/242) had a first PCT concentration of below 1 ng/mL. Nobre and colleagues [20] found that 19.1% of severely septic patients (13/68) had equally low PCT concentrations. Notably, 15% of patients with clinically diagnosed severe sepsis had low PCT concentrations both at study entry and at 72 hours.

PCT concentrations were higher in patients with blood culture-positive severe sepsis, septic shock, or acute renal failure. High PCT concentrations in septic shock or blood culture-positive patients were found in other studies [15,36,37]. Using PCT levels of greater than 0.5 ng/mL as the diagnostic criteria could decrease the need for blood cultures in patients with community-acquired pneumonia by 52% while still identifying 88% of positive cultures [38]. In our more heterogeneous patient population, the PCT concentration cutoff for 88% sensitivity was higher (2.7 ng/mL), with a specificity of 53%. Meisner and colleagues [39] found that higher SOFA scores were associated with higher PCT concentrations in 40 patients, but in our larger study, we found no association with overall organ dysfunction, even with increasing concentrations.

We found higher PCT concentrations in patients with community-acquired infections than in patients with nosocomial infections. Few studies have made comparisons between these patient groups. However, previous sepsis may have an influence on decreasing PCT values compared with patients with primary sepsis [40]. In that study, all cases of secondary sepsis were nosocomial in origin, but 64% of primary sepsis cases were community-acquired. We had significantly more intra-abdominal infections in the nosocomial group; of these patients, 52.9% had ongoing antimicrobial treatment. In general, PCT concentrations may also be influenced by the organism causing infection [41,42].

PCT concentrations in intra-abdominal infections can be useful when deciding the time frame for on-demand laparotomy, and a PCT ratio cutoff value of 1.03 has been proposed to predict successful elimination of the intra-abdominal infection source [43]. In postoperative critically ill patients, the cutoff point for PCT concentration was 1.44 ng/mL to detect worse outcome [44], which may be due to infection and possible unsuccessful control of the source.

In general, the severity of the inflammatory response, the appropriate antimicrobial therapy, the timing for antimicrobial administration, and adequate source control all have influence on infection healing and PCT decrease. These variable factors may explain the differences in PCT concentrations in patients with community-acquired or nosocomial infections.

In our study, unlike in the study by Clec'h and colleagues [15], single PCT concentrations did not predict mortality; however, CRP was equally poor at predicting outcome in both studies. In a French study, the first PCT concentration did not predict outcome, but concentrations were higher in nonsurvivors measured 3 days later [14]. Jensen and colleagues [16] studied the predictive value of PCT in critically ill patients in general and found that concentrations over 1 ng/mL predicted worse outcome. This is in accordance with other studies' cutoff limits that were used to discriminate patients with severe infections from those without severe infections.

In recent studies, a cutoff value of 1 ng/mL was used [20,45] to reduce antibiotic exposure or the length of antibiotic treatment was based on PCT cutoff ranges or decreasing PCT concentrations. In the ProHOSP study, antibiotic administration was strongly encouraged for patients with LRTIs and PCT concentrations of higher than 0.5 ng/mL [18]. Patients in this study had community-acquired pneumonia or LRTI and were not necessarily critically ill [18]. However, in critically ill patients, PCT-guided termination of antibiotic treatment was used without worsening outcome [19,45].

Our study has some limitations. Owing to unavailable consent, blood samples were drawn from only half of the patients (51.2%) in the Finnsepsis study, and ΔPCT could be calculated from only one third of all patients (155/470, 33%). However, the patients with PCT measurements did not differ from the other patients with regard to demographic data or severity of illness. Furthermore, we measured PCT concentrations at only two time points: on the day severe sepsis was diagnosed and 72 hours afterwards, rather than serially during the entire length of stay in the ICU. On the other hand, our study, with 242 patients, is one of the largest published studies of PCT measurements in clinically diagnosed severe sepsis patients who were treated in intensive care. Finally, antibiotic treatment was not adjusted on the basis of PCT, but of clinical response and CRP values. Thus, the outcome was not biased or affected by PCT measurements.

## Conclusions

PCT concentrations are elevated in patients with blood culture-positive infections and septic shock, but single values have no predictive value for patient outcome. However, a decrease in PCT concentrations may be associated with a favorable outcome in patients with severe sepsis. Because of a substantial proportion of severe sepsis patients with low PCT concentrations on admission, clinical suspicion and diagnosis of severe sepsis cannot be replaced with PCT measurements.

## Key messages

• Procalcitonin (PCT) concentrations are elevated in patients with severe sepsis, especially with positive blood culture infections or with septic shock.

• Some patients with severe sepsis may have low PCT levels and the diagnosis cannot be based only on PCT concentrations.

• A substantial decrease in PCT concentration seems to be more important for survival than individual values.

## Abbreviations

APACHE II: Acute Physiology and Chronic Health Evaluation II; AUC: area under the curve; CI: confidence interval; CRP: C-reactive protein; ICU: intensive care unit; IL-6: interleukin-6; IQR: interquartile range; LRTI: lower respiratory tract infection; PCT: procalcitonin; ROC: receiver operating characteristic; SAPS II: Simplified Acute Physiology Score II; SOFA: Sequential Organ Failure Assessment.

## Competing interests

The authors declare that they have no competing interests.

## Authors' contributions

SK contributed the idea and design of the Finnsepsis study and this substudy, analyzed the data, and wrote the initial manuscript. VP and ER contributed the idea and design of the Finnsepsis study and this substudy and contributed to the drafts of the manuscript. EK contributed the idea and design of the Finnsepsis study and this substudy. MH, SV, and KP helped to carry out the analyses and contributed to the manuscript. SA collected the data and contributed to the drafting of the manuscript. All authors read and approved the final version of the manuscript.
